# The economic burden of treating uncomplicated hypertension in Sub-Saharan Africa: a systematic literature review

**DOI:** 10.1186/s12889-022-13877-4

**Published:** 2022-08-08

**Authors:** E. Gnugesser, C. Chwila, S. Brenner, A. Deckert, P. Dambach, J. I. Steinert, T. Bärnighausen, O. Horstick, K. Antia, V. R. Louis

**Affiliations:** 1 Heidelberg Institute of Global Health, Heidelberg University Medical School, Heidelberg University, Heidelberg, Germany; 2 TUM School of Social Sciences and Technology, Technical University of Munich, Munich, Germany

**Keywords:** Hypertension, Sub-Saharan Africa, Economic

## Abstract

**Background and Objectives:**

Hypertension is one of the leading cardiovascular risk factors with high numbers of undiagnosed and untreated patients in Sub Saharan Africa (SSA). The health systems and affected people are often overwhelmed by the social and economic burden that comes with the disease. However, the research on the economic burden and consequences of hypertension treatment remains scare in SSA. The objective of our review was to compare different hypertension treatment costs across the continent and identify major cost drivers.

**Material and Methods:**

Systematic literature searches were conducted in multiple databases (e.g., PubMed, Web of Science, Google Scholar) for peer reviewed articles written in English language with a publication date from inception to Jan. 2022. We included studies assessing direct and indirect costs of hypertension therapy in SSA from a provider or user perspective. The search and a quality assessment were independently executed by two researchers. All results were converted to 2021 US Dollar.

**Results:**

Of 3999 results identified in the initial search, 33 were selected for data extraction. Costs differed between countries, costing perspectives and cost categories. Only 25% of the SSA countries were mentioned in the studies, with Nigeria dominating the research with a share of 27% of the studies. We identified 15 results each from a user or provider perspective. Medication costs were accountable for the most part of the expenditures with a range from 1.70$ to 97.06$ from a patient perspective and 0.09$ to 193.55$ from a provider perspective per patient per month. Major cost drivers were multidrug treatment, inpatient or hospital care and having a comorbidity like diabetes.

**Conclusion:**

Hypertension poses a significant economic burden for patients and governments in SSA. Interpreting and comparing the results from different countries and studies is difficult as there are different financing methods and cost items are defined in different ways. However, our results identify medication costs as one of the biggest cost contributors. When fighting the economic burden in SSA, reducing medication costs in form of subsidies or special interventions needs to be considered.

**Trial registration:**

Registration: PROSPERO, ID CRD42020220957.

**Supplementary Information:**

The online version contains supplementary material available at 10.1186/s12889-022-13877-4.

## Introduction/Background

Hypertension is one of the leading risk factors for numerous non-communicable chronic diseases, such as cardiovascular diseases (CVD) including ventricular hypertrophy and heart failure [1]. The global prevalence of hypertension increased from 594 million in 1975 to 1.13 billion in 2015 with an increase largely in low- and middle income countries (LMICs) [2] and is expected to continue in the future [3, 4]. Today, two thirds of patients with hypertension are living in LMICs [2], which leads to a significant burden considering that most health systems are overwhelmed by the double burden of disease of infectious, communicable (CDs, i.e. Human immunodeficiency virus (HIV), malaria) and chronic, non-communicable diseases (NCDs, i.e. hypertension, diabetes) [5]. Most governments are not yet prepared for treating NCDs with a low number of specific screening and intervention programs leading to high numbers of undiagnosed and untreated patients [6, 7]. Furthermore, while awareness, treatment and control rates related to hypertension in high-income countries (HIC) increased substantially between 2000 and 2010, such increases in awareness and treatment rates have been less substantial in LMICs and have even decreased for hypertension control [8]. Regarding age, in HIC the highest burden of hypertension is found among people aged above 60 years, whereas in LMICs, this burden is highest among the middle-aged (e.g., 40 to 59 years) [8]. Within LMICs, the African region has one of the highest hypertension rates worldwide with a mean prevalence of 57% for adults aged 50 years or older [9–11].

The reasons for these disparities across countries are multi-causal and mostly connected to the increase of cardiovascular risk factors in developing countries, such as rapidly ageing societies, urbanization and life-style changes, such as dietary habits [12]. Additional contributing factors include access and barriers to appropriate medical care, such as the limited availability and affordability of cardiovascular medicines [13, 14]. Furthermore, while the number of patients living with hypertension continues to rise, additional public health challenges (e.g., HIV or Coronavirus disease 2019 (Covid 19) pandemics, recent outbreaks of Ebola, malaria or measles and high maternal morbidity) are often of higher priority and therefore receive more financial and political attention [15]. As a result, economic consequences, such as direct and indirect costs related to the hypertension and its sequelae borne by patients, the health system, and the society at large, add to the already precarious economic situation in some countries.

Assessment of the economic burden of disease is a useful tool for decision-making processes or to reforming public health policies [16]. For instance, previous systematic reviews assessed the economic impact of high blood pressure with respects to the costs incurred by CVD and related complications [17, 18]. Many of these studies, however, focus on HICs only [19]. For LMICs, and especially for Sub-Saharan Africa (SSA), the current evidence remains scarce and inconsistent in terms of cost items and the costing approach used, which poses a barrier for comparison.

Therefore, this systematic review aims to assess the economic burden of hypertension by examining direct and indirect costs incurred by hypertension in SSA countries, and to examine what additional factors influence the economic burden experienced by individuals, the health system, or society at large. Our findings will be especially useful for both policy makers and healthcare providers to identify potential cost drivers to reduce the overall economic burden of hypertension and to identify opportunities for more cost-effective prevention strategies.

## Methods

This systematic review follows the Preferred Reporting Items for Systematic reviews and Meta-analysis (PRISMA) 2020 guidelines [20]. In the following, we describe how we operationalized the recommended methodological steps outlined in these guidelines.

### Literature search

We performed a systematic literature search between 01 October 2020 and 16 October 2020 without a limitation on publication date. An update of the literature search was conducted on 02 January 2022 to identify studies that were published between October 2020 and January 2022. The search was performed in the following databases: PubMed, Web of Science, CINAHL, ISPOR, EconLIT, IBSS and Google Scholar. When selecting search terms, we used terms of three different categories: disease, cost and region. We used a combination of broad search terms such as: ‘hypertension’ and ‘high blood pressure’, ‘economic’, ‘cost’ and ‘expenses’. For the region, we searched for all countries individually [21] as well as with the term ‘sub–Saharan Africa’. Mesh terms were used when applicable. We used the Boolean operator ‘AND’ to combine one term of each category (disease, cost, regional term) with repetition until all possible combinations were achieved. The full search strategy as an example for PubMed can be found in additional file 1.

### Eligibility Criteria

We applied the Population Intervention Comparator and Outcomes (PICOs) criteria for deciding the inclusion and exclusion criteria. We included studies published in English language, referring to patients aged 15 and above, who were diagnosed with hypertension according to the national or international guidelines or were getting prescriptions for antihypertensives. If mentioned, we extracted information on the stage of hypertension according to the NICE guidelines [22]. We also included studies below the common hypertension thresholds (e.g. >115 mmHg Systolic blood pressure), stated by the authors as “elevated blood pressure”, if the cost estimation was conducted for standard treatment of hypertension according to accredited guidelines. We specifically excluded studies on preeclampsia, pulmonary hypertension, secondary hypertension and complications like stroke, chronic heart disease (CHD) or similar events. These conditions were excluded because they are special forms or complication of hypertension and have different treatment approaches and costs. Comorbidities were also not considered to maintain our key focus on hypertension and as they could pose a substantial cost factor adding to standard care [23].

Furthermore, we included studies reporting direct and/or indirect costs incurred by the patient or provider, or any other relevant monetary outcomes (i.e., costs per patient/year, costs as % of gross domestic product (GDP)). All non-monetary outcomes except time lost due to treatment were excluded as well as outcomes describing service or drug prices per unit without the actual quantity used. Time lost due to treatment is an important aspect even if the monetary value is not calculated as it can lead to income or work loss [24].

We considered multiple study designs, excluding literature reviews other than systematic reviews, case reports, commentaries, general correspondences, letters-to-editors, unpublished/non-peer reviewed studies, conference proceedings, and animal studies. There were no restrictions on the time of publication. In order to ensure a more exhaustive search, we screened the references of included articles. Relevant identified studies were exported to Endnote X9 and uploaded to Rayyan Systematic Review Software for further screening [25].

### Study selection

We performed a title and abstract screening to exclude irrelevant studies. We then screened the full text articles against the exclusion/inclusion criteria. Title, abstract, as well as full-text screening were independently performed by two of the authors (EG and CC). Any disagreement was resolved by discussions among all authors.

### Data collection process and Data items

Two authors (EG and CC) independently extracted data by applying a data extraction template (in additional file 2) that was developed specifically for this systematic review.

We extracted data on methodological characteristics of the study such as general information (author, title, publication date, primary outcome), setting (country, region, city, public/ private, hospital/outpatient), as well as study design, data source, time period for data collection, estimation and analytic strategies used, conclusions and limitations. Furthermore, we examined the population characteristics and disease definitions.

To examine economic information, we extracted the type of economic estimates reported, incurred direct medical (activities directly involved with patient management, i.e., medication, laboratory, consultation), direct non-medical (i.e., transportation, food) and indirect costs (i.e., expenses incurred by users due to work or income loss, operating or shared consumable costs incurred by providers) by reported sub-groups. Where applicable, the perspective and methods of the reported costing approach was also recorded.

To compare the different results, the monetary outcomes were converted into US dollar ($) and adjusted to 2021 values using the online tool ‘CCEMG – EPPI-Centre Cost Converter’ seen in other systematic reviews [26] and then converted to represent the monthly costs incurred either by defined cost-bearer and/or by illness episode.

### Risk of bias in individual studies

To evaluate the risk of bias in reported results, two authors (EG and CC) performed a quality assessment independently for each included study. Depending on the reported study type, at least one of the following quality assessment tools was applied: The ‘Consolidated Health Economic Evaluation Reporting Standards (CHEERS) statement’ for cost effectiveness analysis, cost utility, cost benefit analysis and cost minimization studies [27], the ‘National Institutes of Health (NIH)’ quality assessment tool for Before/After intervention studies [28] and the ‘Version 2 of the Cochrane risk-of-bias tool (RoB 2 CRT)’ for Cluster Randomized trials [29]. A study tool based on a concept by Larg et al. [30] and CHEERS was created for cost of illness studies. Any disagreements on study design or quality were resolved by discussion among all authors.

### Summary measures and Synthesis of results

First, results were sorted by time measurement used, i.e., per visit, year, or month. All time period costs were converted to monthly costs. Exceptions were costs for whole clinics or healthcare centers, which were measured per year as well as population costs and lifetime costs for antihypertensive treatments. Second, we disaggregated the results by costing perspective, namely into user/patient and provider/health system perspective. For each result, a detailed description of the costing perspective was provided. Third, we determined the type of visit that the cost calculations were based on, i.e., outpatient, inpatient, emergency, or diagnostic visits. At last, we aggregated and descriptively synthesized extracted information using the following indicators: total cost per patient; direct medical cost per patient including subcategories for reported medication, laboratory, and consultation; direct non-medical costs per patient, including transportation costs; and indirect costs per patient, including monetary loss due to reduced income and productivity from a user perspective, and expenses for operating medical facilities and shared consumables from a provider perspective. However, not all studies reported summaries for the three main outcomes (total, direct, indirect costs) and presented single subcategory results. To facilitate comparison, we divided the results into those reporting summaries for total, direct and indirect costs and those reporting single subcategories. If a study reported both, a summary and subcategory result, it was eligible for comparison in both divisions. Results were presented in form of different graphs and tables.

Additionally, information on factors affecting reported cost and Catastrophic Health expenditure (CHE) information was also synthesized. CHE is defined as the proportion of household income or expenditure spent on healthcare exceeding a certain variable threshold [31]. The threshold is defined by the authors considering the respective living standards, usually between 10–40% of total household expenditure [31]. Data were managed with Microsoft Excel.

## Results

### Literature search

**Fig. 1:**
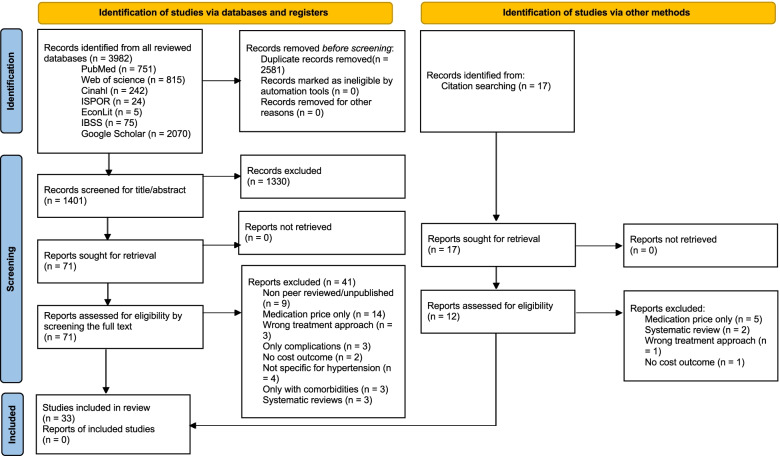
PRISMA flow chart. A flow chart according to the PRISMA guidelines representing the number of studies at each stage of the review

A total of 3982 studies were identified in the initial search process and another 17 were added through searching the reference lists of already included studies. We identified a total 33 studies to include in the final data extraction and analysis process. The numbers of the studies included and excluded at each step, as well as the reasons for exclusion and inclusion are outlined in Fig. 1.

### Study characteristics

#### Design and costing approaches

Study characteristics are summarized in Table 1, a more detailed version can be found in additional file 2. Regarding the economic study design, 21 studies followed a partial economic evaluation (nine cost descriptions [33, 35–38, 44, 47, 49, 56], eight cost analyses [41, 45, 46, 48, 50, 57, 58, 64] and four cost-outcome descriptions [32, 34, 43, 63]. The remaining studies followed a full economic evaluation design (seven cost effectiveness analyses [39, 40, 54, 59–62], three cost utility analyses [42, 51, 52] and two cost minimization analysis [53, 55]). The following costing approaches were used: Bottom up [32–39, 41, 42, 44–49, 51, 54, 55, 58–60, 63], top down [40, 43, 50, 52, 56, 57, 61, 62] and a combination of both [53, 64], human capital [35, 38, 44], replacement value [38] and willingness to pay [46].

**Table 1:** Study characteristics

Study reference	Country	Health economic study design (CA: cost analysis; CEA: cost effectiveness anl., CD: cost description; CMA: cost minimization anl.; COD: Cost outcome description; CUA: cost utility anl.)	Costing approaches (BU: bottom up; HCA: human capital approach; RVA: replacement value approach; WP: willingess to pay; TD: top down)	Costing perspective (CG: caregiver; CO: companion; HO: household; HS: healthsystem; PA: patient; PR: provider, PY: payer; S: societal)	Cost components (0: total cost, 1A: direct medical cost; 1B: direct non medical cost; 2: indirect cost; 3: clinic cost; 4: intervention cost)	Study design generating effectiveness or outcomes (MA: meta analysis, OS: observational study, OT: other study designs, RCT: randomised controlled trial)	Setting (HC: health center; HP: Hospital; MI: missionary; NFP: not for profit organization; NGO: non governmental organization; R: rural; PRI: private; PU: public; U: urban)	Sample definition (AH: arterial hypertension, BP: blood pressure; SPB: systolic blood pressure, y: years)	Sample size (N)	Main outcomes (AH: arterial hypertension, CHE: catastrophic health expenditure, NCD: non communicable diseases)	Quality, (Quality tool used)
Labhardt et al. [32]	Cameroon	COD	BU	PA	1A, 1B	RCT	PU/MI, R, HC	patients diagnosed with uncomplicated AH	130	retention rates and costs of a nurse led intervention	Medium (Rob2)
Dzudie et al. [33]	Cameroon	CD	BU	PA	1A	NA^a^	PU, U, HP	patients diagnosed and treated for AH (≥140/90 mmHg)	408	prescribing patterns of AH drugs and evaluation of effects on BP control	Medium (COI quality)
Lulebo et al. [34]	Dem. Rep Congo	COD	BU	PA + CO	1A, 1B	OS	PU, HP/HC	patients diagnosed with AH (≥140/90)	260	AH control rates and costs of a task shifting intervention	High(CHEERS)
Zawudie et al. [35]	Ethiopia	CD	BU, HCA	PA + CO	0, 1A, 1B, 2	NA^a^	PU/NGO, R/U, HP	stage 1: 140–159/90–99 mmHg, stage 2: ≥ 160/100 mmHg	349	cost of illness (AH)	High (COI Quality)
Bedane [36]	Ethiopia	CD	BU	PA + CG	1A, 1B, 2	NA^a^	PU, U, HP	patients diagnosed with AH	422	out of pocket expenditures for AH patients and caregivers	Medium (COI Quality)
Tolla et al. [37]	Ethiopia	CD	BU	HO	1A, 1B	NA^a^	PU/PRI, U/R, HP	patients diagnosed with AH	235	out of pocket costs and CHE for cardiovascular disease treatment	High (COI Quality)
Adane et al. [38]	Ethiopia	CD	BU, HCA, RVA	PA	0, 1A, 1B, 2	NA^a^	PU, R/U, HP	patients diagnosed with AH	442	cost of illness (AH)	High (COI Quality)
Pozo-Martin et al. [39]	Ghana	CEA	BU	S, PA, PR	0, 1A, 1B, 2, 4	OS	PU/PRI, U, HP/HC	patients treated for AH at least for 12 months, age 18–79 y	10,000 ^c^	evaluation of AH control with a community based intervention	High(CHEERS)
Jha et al. [40]	Guinea	CEA	TD	PR	1A, 3, 4	OT^b^	PU, HC	patients diagnosed with AH	37,100 ^d^	cost and cost effectiveness of an antihypertensive intervention	Medium (CHEERS)
Subramanian et al. [41]	Kenya	CA	BU	PA	1A	NA^a^	PU/PRI, R/U, HP/HC	NR^a^	NR^a^	cost and affordability of different NCDs	High (COI Quality)
Subramanian et al. [42]	Kenya	CUA	BU	HS	1A, 4	MA RCT	NR^a^	cohort with risk index (with BP level and 10 year CVD risk)	1000,000 ^c^	cost and cost effectiveness of a risk stratified mangement approach	High (CHEERS)
Oti et al. [43]	Kenya	COD	TD	PR	1A, 1B, 3, 4	OS	PU/PRI, U, PC	BP ≥ 140/90 mmHg	976	outcomes and costs of a community intervention	Medium (CHEERS)
Oyando et al. [44]	Kenya	CD	BU, HCA	PA + CG	0, 1A, 1B, 2	NA^a^	PU, R, HP/HC	self reported AH, treated for 6 months	212	patient costs for AH treatment	High (COI Quality)
Ba et al. [45]	Mali	CA	BU	PY (insurance)	1A, 1B	NA^a^	PU, HP	patients newly diagnosed with or not yet treated for AH	280	costs in regard to insurance status	High (COI Quality)
Gaziano et al. [46]	Multiple	CA	BU, WP	S	1A, 2, 3	NA^a^	NR^a^	SBP > 115 mmHg	NR^a^	global cost of AH illness and complications	High (COI Quality)
Osibogun et al. [47]	Nigeria	CD	BU	PA	1A	NA^a^	PU, HP	patients diagnosed with AH	147	prescribing patterns and cost of prescription	Medium (COI Quality)
Akunne et al. [48]	Nigeria	CA	BU	PA	1A	NA^a^	PU, HP	patients receiving treatment for AH	1050	prescibing pattern, cost and quality of care	Medium (COI Quality)
Bakare et al. [49]	Nigeria	CD	BU	PA	1A	NA^a^	PU, HP	patients diagnosed with AH	200	prescribing patterns, cost of prescription and laboratory	Medium (COI Quality)
Onwujekwe et al. [50]	Nigeria	CA	TD	HO	1A, 1B	NA^a^	PU/PRI, R/U, HP/HC	AH outpatient and inpatient visits	154	economic burden of different health conditions	High (COI Quality)
Ekwunife et al. [51]	Nigeria	CUA	BU	PR (third party)	1A	MA RCT	NR^a^	cohort with risk index according to 10 year CHD/stroke risk	1000 ^c^	cost effectiveness of drug treatment with different drug classes	High (CHEERS)
Rosendaal et al. [52]	Nigeria	CUA	TD	PR (healthcare)	1A, 4	OS	PU/PRI, R	patients diagnosed with AH (JNC 7 guidelines)	10,000 ^c^	costs and cost effectiveness within an insurance program	High (CHEERS)
Hendriks et al. [53]	Nigeria	CMA	BU, TD	PR (healthcare)	1A, 3	NA^a^	PRI, R, HP	patients diagnosed with AH	322	cost of cardiovascular prevention care in different scenarios	High (CHEERS)
Ilesanmi et al. [54]	Nigeria	CEA	BU	PA	1A, 1B	OS	PU, R, HP	patients diagnosed with AH (JNC 7 guidelines)	250	costs and cost effectiveness of AH treatment	Medium (CHEERS)
Oamen et al. [55]	Nigeria	CMA	BU	PA	1A	OS	PU, HP	patients diagnosed with AH with regular outpatient visits	255	antihypertensive drug use and comparative cost analysis	High(CHEERS)
Eberly et al. [56]	Rwanda	CD	TD	PR (public)	1A, 1B, 3	NA^a^	PU, R, HP	patients diagnosed with AH	223	costs for setting up and maintaining a NCD clinic	Medium (COI Quality)
Ndagijimana [57]	Rwanda	CA	TD	PR (Public)	1A, 1B, 3	NA^a^	PU, R, HP	patients diagnosed with AH	68	cost of providing AH care	Medium (COI Quality)
Bovet et al. [58]	Seychelles	CA	BU	PR	1A	NA^a^	PU, PC	BP stages: 1: 140–159/90–99, 2: > = 160/100	1255	costs for treating high risk cardiovascular disease patients	Medium (COI Quality)
Watkins et al. [59]	South Africa	CEA	BU	PA	1A, 4	MA OS	PU/PRI	NR^a^	1000,000 ^**c**^	cost effectiveness and outcomes of a salt reduction policy	High (CHEERS)
Gaziano et al. [60]	South Africa	CEA	BU	HS	1A, 4	MA OS + RCT	NR^a^	cohort with different guidelines and risk profiles	10,000,000 ^c^	cost and cost effectiveness of different guidelines	High (CHEERS)
Gaziano et al. [61]	South Africa	CEA	TD	PR	1A, 4	MA RCT	U/R, PC	NR^a^	NR^a^	cost and cost effectiveness of a community intervention	High (CHEERS)
Basu et al. [62]	South Africa	CEA	TD	HS	1A	MA OS	NR^a^	BP ≥ 140/90 mmHg or being on AH treatment	7099 ^c^	costs and cost effectiveness of scaling up cardiovascular treatment	High (CHEERS)
Edwards et al. [63]	South Africa	COD	BU	PR (pharmacy)	1A	OS	PU, HC	patients diagnosed and treated for AH	1084	prescribing patterns and costs of new treatment guidelines	Low (Bevor-After)
Settumba et al. [64]	Uganda	CA	BU, TD	PR (public + private)	1A, 1B, 3	NA^a^	PU/PRI NFP, U/R, HP/HC	NR^a^	NR^a^	provider costs of different chronic diseases	High (COI Quality)

^a^*NA* Not applicable, *NR* Not reported

^b^synthesis of several data sources, including RCTs, observational studies, and expert opinions

^c^simulated cohorts

^d^target population

The timeframe for data collection ranged from 20 days to 18 months. For the cost calculation, most studies used medical records [32–35, 38, 45, 47–49, 54–57, 63] or questionnaires and interviews [33–39, 43–45, 50, 64]. Additional sources were price lists [41, 46, 47, 49, 51, 54, 57–59, 62], facility records [34, 39–41, 43, 46, 52, 53, 56, 64] and other literature such as studies on income in South Africa [59, 65] or World Health Organization (WHO) reports [40, 43, 59, 66].

We identified convenient sampling [33–35, 38, 45, 48, 53, 56, 63] as the most used sampling method followed by random sampling [32, 36, 49, 50, 55, 58].

#### Geographical context and setting

**Fig. 2:**
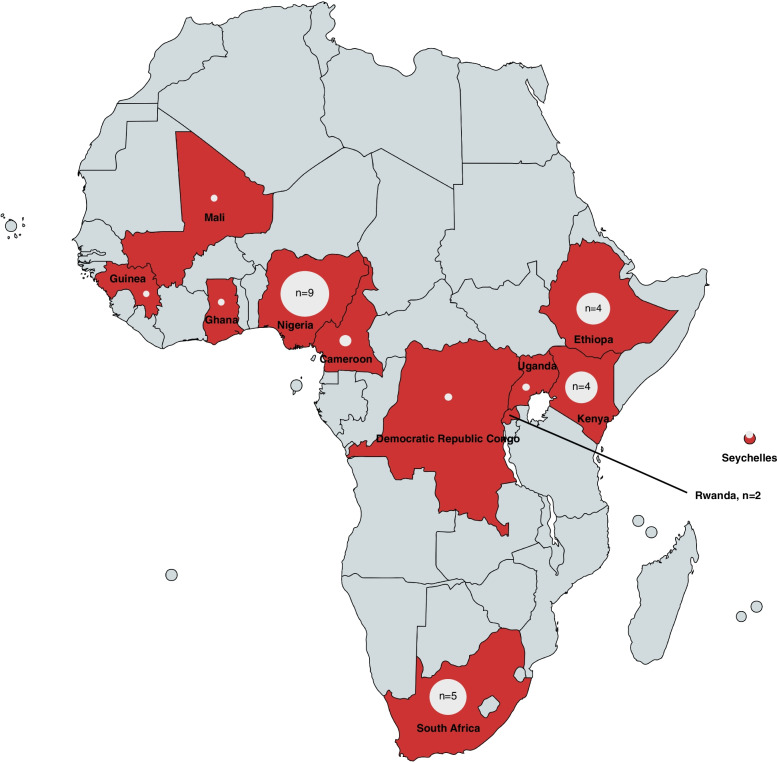
Map of researched countries in Sub Saharan Africa. A map of researched countries in Sub Saharan Africa. The number of studies reported was presented with sizeable dots

Figure 2 displays the countries with research on economic burden of hypertension. Nine studies were conducted in Nigeria [35–38, 47–55], five in South Africa [59–63], four in Kenya [41–44], four in Ethiopia [35–38], two in Rwanda [56, 57] and Cameroon [32, 33] and one each in the Democratic Republic of Congo [34], Ghana [39], Guinea [40], Mali [45], the Seychelles [58] and Uganda [64]. Overall, we identified ten studies [34–38, 40, 45, 56, 57, 64] from low-income countries (LIC) according to the World Bank classification [21] as well as 22 studies [32, 33, 39, 41–44, 47–55, 58–63] from middle income countries (MIC). One study had a global focus [46]. Most studies focused on a rural setting [32, 44, 52–54, 56, 57] or had a mixed urban-rural focus [35, 37, 38, 41, 50, 61, 64] with data collected from hospitals [33, 35–38, 45, 47–49, 53–57], health care or community centers [32, 40, 43, 58, 61, 63] and a mix of facility types [34, 39, 41, 44, 50, 64].

#### Hypertension definition

We found that the definition of hypertension was heterogeneous. Thirteen studies only considered patients diagnosed with hypertension [32, 34, 36–38, 40, 45, 47, 49, 53, 55–57], five studies included only patients who were actually receiving some form of treatment for hypertension [33, 39, 44, 48, 63], and five studies did not provide any specific information other than referring to patients living with hypertension or hypertensive disease [41, 50, 59, 61, 64].

#### Participant characteristics

Participant characteristics varied across the included studies. All patients were aged 15 years and older with the mean age ranging from 48 to 61 years. Overall, more women (56%) than men participated across the studies. The sample size ranged from 68 to 1255 with a cumulative sample size of 8522. However, for some studies authors used simulated cohorts ranging from 1000 to the cumulative number of patients with hypertension in SSA with several million people.

#### Costing perspective

Most studies focused on the public sector [33, 34, 36, 38, 40, 44, 45, 47–49, 54–58, 63] or used a mixed approach also including private, non-governmental or missionary facilities [32, 35, 37, 39, 41, 43, 50, 52, 59, 64]. Only one study had a sole focus on the private health system sector [53].

For better comparison, we categorized reported costs further into those incurred by users or patients [32–39, 41, 44, 45, 47–50, 54, 55, 59] and those incurred by providers [39, 40, 43, 51–53, 56–58, 61, 63, 64] or the broader health system [39, 42, 46, 60, 62]. A detailed description of the costing perspective of each result can be found in Table 1.

#### Cost measurements

We found five studies that evaluated costs per visit [41, 52, 55, 61, 64], four that evaluated recurrent and capital costs to clinic providers [39, 56, 57, 64] and 30 that reported costs per a specific time period like month or year [32–45, 47–54, 56–63]. We determined the type of visit that the cost calculations were based on, which were either outpatient visits [32–37, 39–44, 47–64], inpatients visits [37, 44, 50] or an average of different visit types [37–39, 44, 46, 50]. Five studies reported results for special visit types like diagnostic or screening visits [39, 41, 44, 52], medicine collection visits [39, 44] and unplanned emergency visits [44] or outpatient visits for newly diagnosed patients only [45].

There were 15 studies addressing a user or payer perspective [32–39, 41, 44, 45, 47–50, 54, 59]. The described subcategories for direct medical costs were medications [32–36, 38, 41, 44, 45, 47–49, 54], laboratory services [34–36, 38, 44, 49], consultations [34, 44, 45] and other expenditures like administration or registration [35, 36]. For direct non-medical costs, transportation [32, 34–36, 38, 44, 45, 50, 54], food [34, 35, 44] and other costs for sports and accommodations [35, 44, 45] were stated. Indirect costs were addressed in four studies [35, 38, 39, 44].

We identified 15 studies addressing costs from the provider or health system perspective [39, 40, 42, 43, 46, 51–53, 56–58, 60–64]. The reported subcategories were costs for consultation [39, 53, 62], laboratory [39, 53, 56, 61, 62] and medication [39, 42, 51, 53, 56, 58, 61–63]. Four studies presented annual costs for installing and operating clinics in the context of outpatient hypertension treatment with direct and indirect costs [39, 56, 57, 64].

Cost-effectiveness or other cost-effect outcomes were assessed in twelve studies [32, 34, 39, 40, 42, 43, 52, 53, 55, 59–63].

Apart from the cost calculations, we also found several additional outcomes like affordability of treatment as percentage of monthly income [41, 44, 47, 49, 54] or information on catastrophic household expenditure (CHE) and poverty caused by hypertension treatment costs [35, 37, 41, 44, 59].

### Risk of Bias/study quality

With regards to study quality, we rated 20 studies as high [34, 35, 37–39, 41, 42, 44–46, 50–53, 55, 59–62, 64], twelve as medium quality [32, 33, 36, 40, 43, 47–49, 54–57] and one as low quality [63]. While the study designs itself varied in terms of quality [67], we found that none of the cost-of-illness studies described a sensitivity analysis. Another reason for poor quality was the lack of information on missing data and excluded participants at each stage of the research process. A detailed description can be found in additional file 3.

### Economic outcomes

**Fig. 3:**
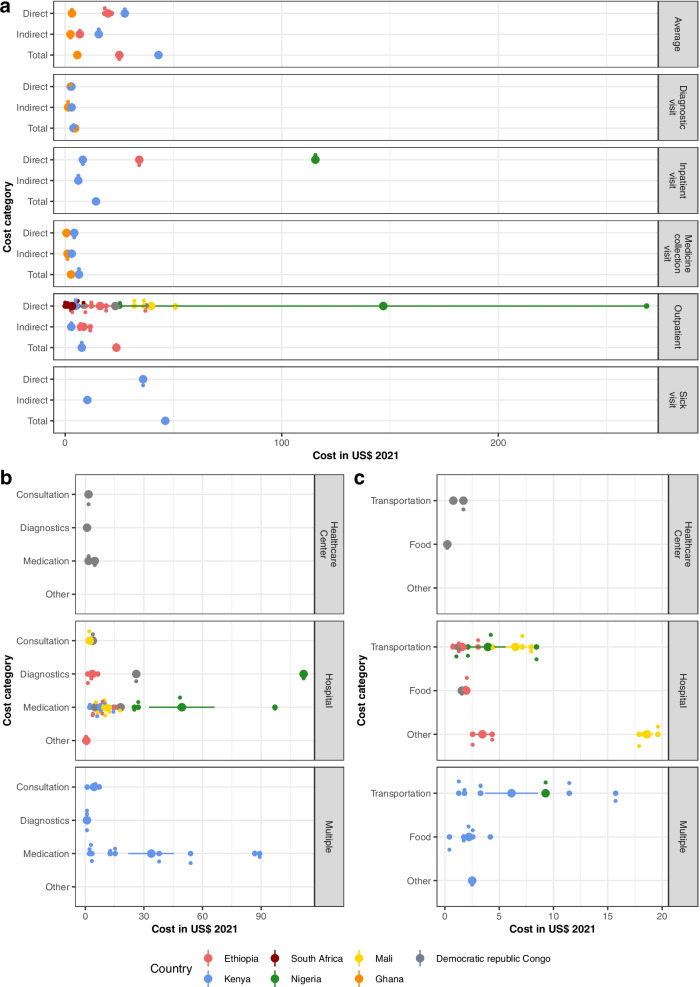
Costs from a patient and user perspective (US$ 2021) per month by cost category. Cost categories were divided into summary costs (Panel a) and the subcategories direct medical (Panel b) and direct non-medical costs (Panel c), displayed by visit type (Panel a), setting (Panel b, c) and country. Each circle in the box plot represents an individual result with bigger circles the mean cost in the subcategory by country and the 95% Confidence Interval (vertical lines)

In Fig. 3, we display the monthly summary and subcategory cost from a patient and user perspective, sorted by visit types and settings. Panel a) shows the summary costs for antihypertensive treatment ranging from 3.84$ for diagnostic visits to 46.24$ for unplanned emergency visits per month [44]. Direct costs from a patient perspective ranged from 0$ in a free healthcare setting in South Africa [59] to 37.80$ per month in an outpatient hospital setting in the Democratic Republic Congo [34]. Highest costs were reported from a household perspective for outpatient treatment in Nigeria with an amount of 268.48$ per month followed by inpatient treatment, also reported from a household perspective (115.56$) [50]. For indirect costs, the lowest cost was 2.83$ per month for outpatient visits while the highest cost was at 15.52$, which was assessed as an average for different visit types [44].

For the subcategory results shown in panel b), most authors reported medication costs with a range from 1.70$ in a health center in Cameroon [32] to 97.06$ per month in a hospital in Nigeria [47]. The most significant differences between medication costs were examined for different countries, showing that Nigeria reported the highest costs [47–49, 54]. Overall, laboratory and consultation costs were lower than medication costs except for one result from Nigeria, which reported monthly laboratory costs of up to 111.75$ [49].

The results for direct non-medical cost categories were dominated by transportation costs, which ranged from 0.74$ per month for outpatient treatments in Ethiopia [36] to 15.72$ for unplanned emergency visits in Kenya [44]. Costs mostly depended on visit type and frequency, with higher costs for unplanned [44] and more frequent visits [54] as well as being treated in hospitals rather than healthcare centers.

In addition to the monetary values, three authors reported indirect cost as time lost due to hypertension treatment. The results were an average monthly loss of 1.3 to two working days for patients as well as one day for the caregiver [35, 36]. Additionally, one author reported that 30% of the study participants missed a median of 17 days over the last three months and 42% were suffering from disturbed social life, including job loss or divorce [44].

**Fig. 4:**
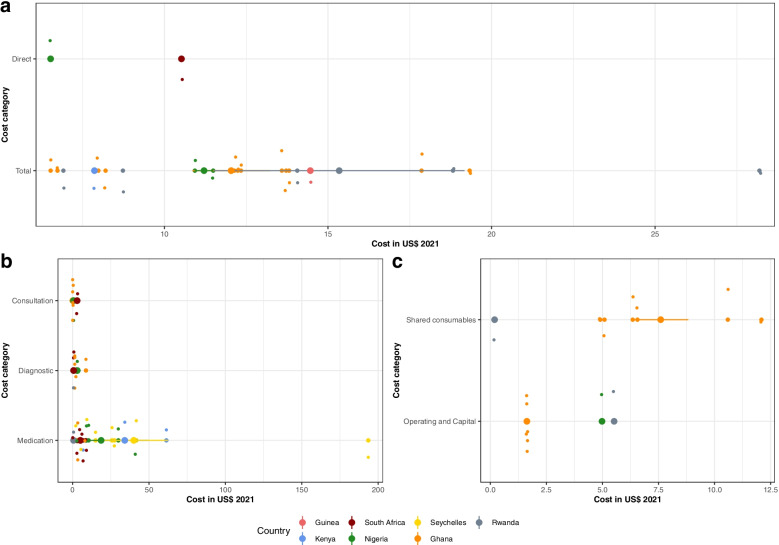
Monthly costs from a provider perspective by cost category. Cost categories were divided into summary costs (Panel a) and the subcategories direct (Panel b) and indirect costs (Panel c), displayed by country. Each circle in the box plot represents an individual result with bigger circles the mean cost in the subcategory by country and the 95% Confidence Interval (vertical lines)

Results from a provider or broader perspective (Fig. 4) were similarly divided into summary costs and subcategory results with the summary costs ranging 6.91$ per month for regular outpatient treatment [56] to 28.19$ for stage three hypertension outpatient treatment [57] from a public provider perspective in Rwanda (panel a).

In panel b), medication costs were highest compared to consultation and laboratory costs, with substantial differences between countries with different health systems and financing structures. The expenditures were as low as 0.09$ per month for outpatient, betablocker based treatment in South Africa from a health system perspective [62], whereas the maximum cost was reported in the Seychelles from a provider perspective for outpatient hypertension treatment with four antihypertensives (193.55$, 2004) before a drug reform took place [58]. The reform led to the use of lower priced medications and therefore a lower overall cost (27.30$, 2005) [58]. Panel c shows indirect provider costs in the form of operating and shared consumables cost. Despite the difference between the countries, costs for consumable products were overall higher than operating costs for maintaining buildings and machines.

**Fig. 5:**
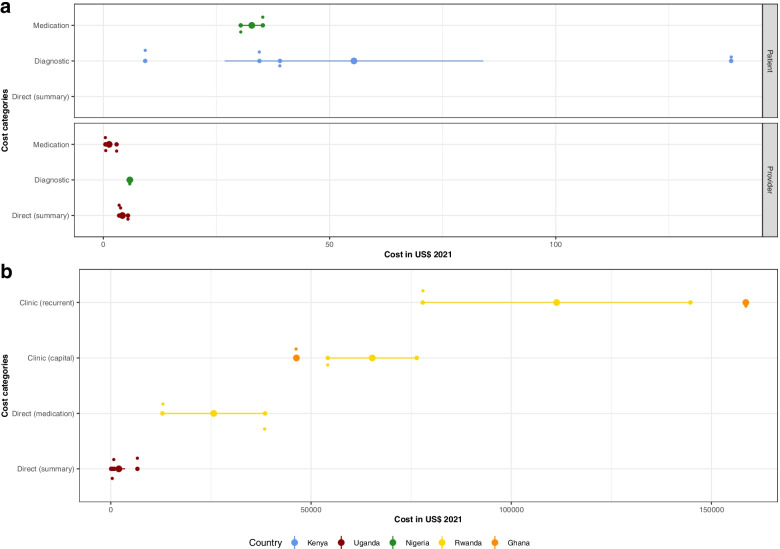
Economic outcomes per episode and clinic costs. Cost categories were divided into Costs per episode (Panel **a**) and costs for clinics per year (Panel **b**), displayed by country. Each circle in the box plot represents an individual result with bigger circles the mean cost in the subcategory by country and the 95% Confidence Interval (vertical lines)

Figure 5 depicts the costs per episode, e.g. single visits, and costs for clinics in form of capital and recurrent costs as well as total service costs per year. Overall, costs per episode were higher from a patient perspective than a provider perspective. When looking at the clinic costs, data from two hospitals in Rwanda showed that capital costs were lower than recurrent costs. Medication was the most dominant cost component, representing 27% [57] and 17% [56] of the costs respectively. In a study from Uganda [64], the authors stated that hospitals had a much higher total service cost per year than healthcare centers, especially level four, nurse-led healthcare centers. One study reported the lifetime costs for treating hypertension in South Africa, which amounted up to 2497.24$ from a provider perspective [61].

Macroeconomic costs ranged from $1.64million annually for the full population of patients ≥25 years living with hypertension on the Seychelles [58] to $397.64 million for direct costs for hypertensive treatment in the SSA population with systolic blood pressure ≥ 115 mmHg [46]. The latter reported costs drawing on population data from the Global Burden of disease project [68] with indirect costs ranging from $10 to $32 billions of potential welfare losses when using 30 times or 100 times GDP per capita when assigning monetary values to production losses.

### Cost drivers

In Table 2, we displayed the thirteen studies reporting drivers of costs. In Nigeria, having a comorbidity [47, 53, 54] like diabetes, renal or heart disease increased the expenditure. In contrast, a lower number of drugs, a lower stage of disease, controlled hypertension or spending <10% of household income on treatment reduced the overall payments [54]. In Ethiopia, inpatient admission [37, 38], higher family size [35], higher distance from the hospital [35], presence of a companion [35] or hypertension in a later stage [35], multidrug treatment [38], highest socioeconomic status [38], college or above education [38], being a government employee [38] or having a comorbidity [38] increased costs. Conversely, having primary education compared to no education [35] and being retired [38] was associated with lower cost.

**Table 2:** Cost drivers

	Item (Reference)	Statistical test with value (***p*** value or effect size), (BLR: bivariate linear regression, GLM: general linear model, CS: Chi square test, FE: Fisher’s exact test)
**Positive Effect (higher costs)**	• General Hospital [34] • Hospital, Health Centers (HC) Level 3 [64] • Private facilities [41]	• NR^a^ • NR^a^ • NR^a^
	• Family size (4–6 people) [35] • Family size (>6 people) [35]	• BLR (ß-Coefficient adjusted: 0.107 (0.044, 0.171), unadjusted: 0.122 (0.050, 0.195)) • BLR (ß-Coefficient adj.: 0.115 (0.044, 0.186), unadj.: 0.122 (0.050, 0.195))
	Higher Distance from hospital [35]	BLR (ß-Coefficient adj.: 0.003 (0.002, 0.004), unadj.: 0.003 (0.002, 0.004))
	Presence of a companion [35]	BLR (ß-Coefficient adj.: 0.096 (0.057, 0.135), unadj.: 0.106 (0.064, −0.149)
	• Hypertension stage 2 [35] • Higher number of antihypertensives or stage [41] • Multidrug treatment [38]	• BLR (ß-Coefficient adj.: 0.070 (0.023, 0.118), unadj.: 0.074 (0.021, 0.126)) • NR^a^ • GLM (Exp(b) = 1.32, *p* < 0.001)
	• Inpatient admission [37] • Hospitalization [38]	• NR^a^ • GLM (Exp(b) = 1.87, *p* < 0.001)
	• Highest Socioeconomic status (SES) [38] • Higher income quintiles [59]	• GLM (Exp(b) = 1.4, *p* < 0.001) • NR^a^
	• Education college and above [38] • Government employment [38]	• GLM (Exp(b) = 1.35, *p* = 0.016) • GLM (Exp(b) = 1.30, *p* = 0.012)
	• plus Comorbidity [38, 47, 54] • plus diabetes [47, 53] • plus heart disease [47] • plus renal disease [47]	• GLM (Exp(b) = 1.20, *p* = 0.04), FE (34.940, *p* = <0.001), T test (2.899, *p* = 0.004) • FE (8.879, *p* = 0.012), NR^a^ • FE (20.082, *p* = 0.001) • FE (6.673, *p* = 0.030)
	Sick visit (compared to average) [44]	NR^a^
	• plus Insurance (total cost) [45] • plus insurance (cardiovascular medication) [45] • plus Insurance (Consultation) [45]	• CS (*p* < 0.0001) • CS (*p* < 0.0001) • CS (*p* = 0.018)
**No effect**	Age [35, 38, 54], gender [35, 38, 54], secondary / tertiary education [35, 38], marital status [35], residence (urban/rural) [35], stage (prehypertension to stage 1) [35], duration of illness [35], plus comorbidity [35], plus dyslipidemia [47], plus complications [38], occupation farmer [38], middle SES [38], income group [54], persistence to therapy [54], plus insurance (Transport, Chest x ray, Blood test, Echocardiography, other, non-cardiovascular medication) [45]	
**Negative effect (reduced costs)**	• HC [34] • HC level 2, level 4 [64]	• NR^a^• NR^a^
	Primary education (vs no education) [35]	BLR (ß-Coefficient adj.: −0.072 (−0.0124, −0.020), unadj.: −0.068 (−0.126, −0.009))
	Retirement [38]	GLM (Exp(b) = 0.71, *p* = 0.001)
	Diagnostic visit, medicine collection, scheduled visit, inpatient admission (compared to average) [44]	NR^a^
	Plus insurance (Electrocardiogram) [45]	CS (*p* = 0.001)
	• Lower stage [54] • Lower number of antihypertensives [54] • Generic Ramipril (vs branded) [55]	• T-test (4.689, *p* < 0.001) • T-test (21.313, *p* < 0.001) • T-test (4.54, *p* = 0.005)
	Controlled Hypertension [54]	T-test (2.618, *p* = 0.009)
	% of household income spent on treatment <10% (compared to ≥10% spent) [54]	T-test (12.719, *p* < 0.001)

^a^*NR* Not reported

In Kenya, different visit types were compared showing that sick visit costs were higher compared to the average visit costs and or other visit types [44]. Additionally, costs were found to be higher in private facilities and increased with the number of drugs and hypertension stage [41]. In Mali, having an insurance affected the costs among patients with hypertension [45]. Total cardiovascular medication and consultation cost were higher with the insurance. Two studies from Uganda [64] and the Democratic Republic of Congo [34] compared different health facility types and found that costs were higher in hospitals than in health centers. One study from South Africa [59] also reported higher costs associated with higher income quintile of the patient.

A study from Ghana evaluated the effect of replacing brand medications for Ramipril with the generic drug, which led to significantly lower costs [39].

### Catastrophic health expenditure/affordability

Several authors reported information on catastrophic health expenditure (CHE), which was mostly defined as an expenditure on hypertension treatment of ≥10% of the monthly income (see Table 3). We found that CHE was a substantial problem across different countries with up to 72% of patients in Ethiopia being affected [35]. A detailed description can be found in Table 3.

**Table 3:** Catastrophic Health expenditure (CHE) among hypertensive patients

CHE % (Study reference)^a^	Effects on CHE (Study reference)			% of income spent on treatment (Study reference)	
• 72% [35]^b^ • 59% [44]^c^ • 52.8% [54] • 43.3% [44]^h^ • 42% [35]^c^ • 26.7% [37]^d^ • 7.9% [37]^e^ • 6.54% [41]^f^	Higher risk: • Rural settings (62%) [35] • No education (62%) [35] • Hospitalization [37] • Private hospital [37] • Rural setting [37] • Developing complications (stroke) [37] • High Family size [37] • Lower income [37, 41, 44]			• 1–2% (public sector) [41] • 8–10% (private sector) [41] • 11,1% [54] • 11,4% [49] • 12,9% [37]^d, i^ • 17,6% [37]^e, i^ • 36,7% [47]	
	Lower risk: • Urban setting (29%), [35] • Primary (28%), secondary (34%) or tertiary (17%) education [35] • Longer duration since diagnosis [37] • Higher income [37]				
	No significant change: • Age [37] • Occupation [37]				
**Effects by income quintile (Q1-Q5, lowest to highest)**					
Income quintile	Q1	Q2	Q3	Q4	Q5
CHE %	• 8.0% [37]^e^ • 8.7% [41]^f^ • 27.9% [37]^d^ • 33.6% [44]^g^	• 7.1% [37]^e^ • 7.3% [41]^f^ • 25.6% [44]^g^ • 28.5% [37]^d^	• 7.3% [41]^f^ • 9.3% [37]^e^ • 14.4% [44]^g^ • 32.2% [37]^d^	• 5.6% [41]^f^ • 7.7% [37]^e^ • 15.2% [44]^g^ • 28.3% [37]^d^	• 3.8% [41]^f^ • 7.7% [37]^e^ • 11.2% [44]^g^ • 13.9% [37]^d^
% of income	• 23.6% [37]^d^ • 14.5% [37]^e^	• 23.9% [37]^d^ • 25.2% [37]^e^	• 14.0% [37]^d^ • 9.3% [37]^e^	• 12.9% [37]^d^ • 9.3% [37]^e^	• 4.8% [37]^d^ • 3.0% [37]^e^

^a^CHE defined as annual expenditure ≥10% of annual household income

^b^for direct + indirect costs

^c^for direct costs

^d^financed by income only

^e^financed by income + others (e,g. family, insurance, etc.)

^f^CHE defined as ≥40% of non food expenditure

^g^Among CHE respondents

^h^excluding costs for transportation

^i^ mean positive overshoot >10% income

## Discussion

To our knowledge, this is the first systematic review focusing on economic burden of hypertension in SSA. Our review found that treatment of hypertension poses a great economic burden for many people and providers in SSA, with high expenditures compared to monthly household income and wages. We were able to identify research gaps and major cost drivers as important target points for future interventions.

Our analysis showed that costs differed substantially depending on costing perspectives, healthcare facility setting and type of visit for in- and outpatient admissions. Therefore, it was difficult to examine overall trends across whole SSA with each country posing different premises in terms of health system infrastructure, financing, and even cultural differences. However, major cost drivers identified across multiple countries were comorbidities, being treated in hospitals or private health institutions and higher socioeconomic status.

In 2015, Gheorghe et al. [17] argued that the mean direct costs for hypertension was Int$ 22 per month in low- and middle-income countries which is in the same range as our results. While this cost may be considered as an average across LMIC settings, our research shows that costs across health care systems differ substantially; The lowest cost estimate in our systematic review represents merely a tenth of the cost reported in Gheorghe et al., whereas our highest estimate is more than ten times higher than the average costs in this previous study.

In contrast to our findings, hypertension treatment costs were found to be much higher in western countries. For example, in a study conducted in the US [69], the estimated mean annual hypertension-health expenditures were US $3914 – and thus more than eleven times higher than in our review. Overall, the costs were also higher for per person outpatient payments [70] compared with up to 40 times lower in South Africa. However, studies from Ethiopia and Nigeria reported equal or even slightly higher costs than the US. Consistent to our research, a study from Burkina Faso reported monthly costs of US $11.5 for hypertensive patients in 2015 with 66.9% representing medication costs of the overall costs [71]. A study from Nigeria confirmed these findings with monthly costs of US $44.35 with medication costs representing more than half of the overall costs [72].

One reason for discrepancies could be that the costs of living, goods and services differ across the globe due to different taxation systems or the availability of generics [73]. Additionally, the “Penn-effect” could play a role, which describes differences in price levels for goods and services across countries linear their per capita income. Therefore, the consumer prices are higher in HIC than in LMICs. This effect is based on assumptions from Balassa-Samuelson that describe the association of an economy’s productivity growth in the traded goods sector with wage and price levels in the non-traded or service sector [74, 75].

### Recommendations and implications

Our review did not identify any empirical evidence on hypertension costs from some of the poorest countries on the continent. Only 25% of the SSA countries presented any research. In addition, fine-grained data on indirect costs and costs of implementation of clinics and community health centers are lacking. These data gaps need to be filled in future research endeavors.

Our study found that private, hospital and rural care tend to be more expensive and had a negative effect on CHE. This might be explained by lower awareness and control rates among rural populations [10, 76, 77], more severe cases treated in hospitals as well as more people with higher socioeconomic status using private health systems [78]. In addition, we found that CHE and low affordability were the main barriers of access to needed care. Hence, incentives or free medication packages need to be considered alongside other targeted interventions. This would also help to improve accessibility, affordability and adherence [79], which, in turn, could further reduce future healthcare expenditure [80].

Our recommendation for future researchers and policy makers are therefore to focus on more countries in SSA with a special consideration of indirect costs and cost of implementation of clinics and community centers. We further recommend lowering the cost of medications especially when paid out of pocket by the patients.

### Strength and Limitations

By applying a systematic approach, we were able to include cost data from different cost perspectives and designs without any limitation on the timeframe or clinic setting. Additionally, we assessed the major drivers of costs, which highlights important target points for interventions to alleviate the economic burden of hypertension.

Several limitations of our study should be mentioned. First, although we used a systematic approach for identifying relevant articles through multiple databases with different search terms, there is a possibility of missing some relevant studies. Second, the majority of studies did not state a clear definition of hypertension and rather used terms like “patients diagnosed with hypertension” or “patients receiving antihypertensive treatments”. When one of the above was stated, we assumed that the authors were referring to a national or international guideline for the definition of hypertension. However, this could pose a possible risk of bias by including ineligible patients and studies. The heterogeneous nature of the outcome measures used in the studies made it impossible to perform a meta-analysis. Considering that included studies assessed direct costs in different ways, the results are difficult to interpret for this cost category. We found that splitting costs into different categories with a focus on the medication and treatment costs may help to address this problem.

## Conclusions

Our results show that in SSA, hypertension treatment poses an economic burden, in which, medication cost and indirect costs contribute significantly. The systematic review highlighted the lack of research on hypertension costs in the poorest countries of SSA. Although, it is difficult to provide recommendations that would be equally effective for all countries included in this study, our analysis showed, that subsidizing drugs could lead to the most substantial reduction in treatment costs. A further reduction in expenditure could be achieved by promoting treatment in local health and community centers rather than hospitals.

## Supplementary Information

**Additional file 1.** Example search string for PubMed.

**Additional file 2.** Data extraction template.

**Additional file 3.**

## Data Availability

Data sharing is not applicable to this article as no datasets were generated or analysed during the current study.

## Abbreviations

CD: Communicable Disease
CHD: Chronic heart disease
CHE: Catastrophic Health expenditure
CHEERS: Consolidated Health Economic Evaluation Reporting Standards statement
CVD: Cardiovascular disease
GDP: Gross domestic product
HIC: High-Income countries
HIV: Human immunodeficiency virus
LIC: Low-income countries
LMIC: Low- and Middle-income countries
MIC: Middle-income countries
NCD: Non communicable disease
NIH: National Institutes of Health
PICO: Population Intervention Comparator and Outcomes criteria
PRISMA: Preferred Reporting Items for Systematic reviews and Meta-analysis
Rob2: Version 2 of the Cochrane risk-of-bias tool
SSA: Sub Saharan Africa
WHO: World Health Organization
