# Supplementary material for: The economic burden of treating uncomplicated hypertension in Sub-Saharan Africa: a systematic literature review
Source: BMC Public Health. 2022 Aug 8;22:1507. doi: 10.1186/s12889-022-13877-4 (PMC9358363; doi:10.1186/s12889-022-13877-4)
Supplement: Supplementary file 1 — Additional file 1. Example search string for PubMed. [file 12889_2022_13877_MOESM1_ESM.docx]

Additional File 1: Example search string for PubMed

*An example of the search string and Boolean operators used in the search. The search string was the same for all databases. Here, an example is given for PubMed.*

Search PubMed (-Jan.02 2022), Date of search: Oct.1 2020, Jan.2 2022 (update)

| Search term #1 | Search term #2 | Search term #3 | Hits |
| --- | --- | --- | --- |
| (Economic) AND | (High blood pressure) AND | (Sub Saharan Africa) OR (Angola) OR (Benin) OR (Botswana) OR (Burkina Faso) OR (Burundi) OR (Cabo Verde) OR (Cameroon) OR (Central African Republic) OR (Chad) OR (Comoros) OR (Congo) OR (Democratic Republic Congo) OR (Cote d’Ivoire) OR (Ivory Coast) OR (Djibouti) OR (Equatorial Guinea) OR (Eritrea) OR (Ethiopia) OR (Gabon) OR (Gambia) OR (Ghana) OR (Guinea) OR (Guinea-Bissau) OR (Kenya) OR (Lesotho) OR (Liberia) OR (Madagascar) OR (Malawi) OR (Mali) OR (Mauritania) OR (Mauritius) OR (Mozambique) OR (Namibia) OR (Niger) OR (Nigeria) OR (Reunion) OR (Rwanda) OR (Sao Tome and Principe) OR (Senegal) OR (Seychelles) OR (Sierra Leone) OR (Somalia) OR (South Africa) OR (Sudan) OR (Swaziland) OR (Tanzania) OR (Togo) OR (Uganda) OR (Zambia) OR (Zimbabwe) or (East Africa) OR (Middle Africa) OR (Southern Africa) OR (West Africa) OR (Central Africa) OR (Western Sahara) | 30 |
| (Economic) AND | (Hypertension) AND | (Sub Saharan Africa) OR (Angola) OR (Benin) OR (Botswana) OR (Burkina Faso) OR (Burundi) OR (Cabo Verde) OR (Cameroon) OR (Central African Republic) OR (Chad) OR (Comoros) OR (Congo) OR (Democratic Republic Congo) OR (Cote d’Ivoire) OR (Ivory Coast) OR (Djibouti) OR (Equatorial Guinea) OR (Eritrea) OR (Ethiopia) OR (Gabon) OR (Gambia) OR (Ghana) OR (Guinea) OR (Guinea-Bissau) OR (Kenya) OR (Lesotho) OR (Liberia) OR (Madagascar) OR (Malawi) OR (Mali) OR (Mauritania) OR (Mauritius) OR (Mozambique) OR (Namibia) OR (Niger) OR (Nigeria) OR (Reunion) OR (Rwanda) OR (Sao Tome and Principe) OR (Senegal) OR (Seychelles) OR (Sierra Leone) OR (Somalia) OR (South Africa) OR (Sudan) OR (Swaziland) OR (Tanzania) OR (Togo) OR (Uganda) OR (Zambia) OR (Zimbabwe) or (East Africa) OR (Middle Africa) OR (Southern Africa) OR (West Africa) OR (Central Africa) OR (Western Sahara) | 371 |
| (Cost) AND | (High blood pressure) AND | (Sub Saharan Africa) OR (Angola) OR (Benin) OR (Botswana) OR (Burkina Faso) OR (Burundi) OR (Cabo Verde) OR (Cameroon) OR (Central African Republic) OR (Chad) OR (Comoros) OR (Congo) OR (Democratic Republic Congo) OR (Cote d’Ivoire) OR (Ivory Coast) OR (Djibouti) OR (Equatorial Guinea) OR (Eritrea) OR (Ethiopia) OR (Gabon) OR (Gambia) OR (Ghana) OR (Guinea) OR (Guinea-Bissau) OR (Kenya) OR (Lesotho) OR (Liberia) OR (Madagascar) OR (Malawi) OR (Mali) OR (Mauritania) OR (Mauritius) OR (Mozambique) OR (Namibia) OR (Niger) OR (Nigeria) OR (Reunion) OR (Rwanda) OR (Sao Tome and Principe) OR (Senegal) OR (Seychelles) OR (Sierra Leone) OR (Somalia) OR (South Africa) OR (Sudan) OR (Swaziland) OR (Tanzania) OR (Togo) OR (Uganda) OR (Zambia) OR (Zimbabwe) or (East Africa) OR (Middle Africa) OR (Southern Africa) OR (West Africa) OR (Central Africa) OR (Western Sahara) | 29 |
| (Cost) AND | (Hypertension) AND | (Sub Saharan Africa) OR (Angola) OR (Benin) OR (Botswana) OR (Burkina Faso) OR (Burundi) OR (Cabo Verde) OR (Cameroon) OR (Central African Republic) OR (Chad) OR (Comoros) OR (Congo) OR (Democratic Republic Congo) OR (Cote d’Ivoire) OR (Ivory Coast) OR (Djibouti) OR (Equatorial Guinea) OR (Eritrea) OR (Ethiopia) OR (Gabon) OR (Gambia) OR (Ghana) OR (Guinea) OR (Guinea-Bissau) OR (Kenya) OR (Lesotho) OR (Liberia) OR (Madagascar) OR (Malawi) OR (Mali) OR (Mauritania) OR (Mauritius) OR (Mozambique) OR (Namibia) OR (Niger) OR (Nigeria) OR (Reunion) OR (Rwanda) OR (Sao Tome and Principe) OR (Senegal) OR (Seychelles) OR (Sierra Leone) OR (Somalia) OR (South Africa) OR (Sudan) OR (Swaziland) OR (Tanzania) OR (Togo) OR (Uganda) OR (Zambia) OR (Zimbabwe) or (East Africa) OR (Middle Africa) OR (Southern Africa) OR (West Africa) OR (Central Africa) OR (Western Sahara) | 316 |
| (Expenses) AND | (High blood pressure) AND | (Sub Saharan Africa) OR (Angola) OR (Benin) OR (Botswana) OR (Burkina Faso) OR (Burundi) OR (Cabo Verde) OR (Cameroon) OR (Central African Republic) OR (Chad) OR (Comoros) OR (Congo) OR (Democratic Republic Congo) OR (Cote d’Ivoire) OR (Ivory Coast) OR (Djibouti) OR (Equatorial Guinea) OR (Eritrea) OR (Ethiopia) OR (Gabon) OR (Gambia) OR (Ghana) OR (Guinea) OR (Guinea-Bissau) OR (Kenya) OR (Lesotho) OR (Liberia) OR (Madagascar) OR (Malawi) OR (Mali) OR (Mauritania) OR (Mauritius) OR (Mozambique) OR (Namibia) OR (Niger) OR (Nigeria) OR (Reunion) OR (Rwanda) OR (Sao Tome and Principe) OR (Senegal) OR (Seychelles) OR (Sierra Leone) OR (Somalia) OR (South Africa) OR (Sudan) OR (Swaziland) OR (Tanzania) OR (Togo) OR (Uganda) OR (Zambia) OR (Zimbabwe) or (East Africa) OR (Middle Africa) OR (Southern Africa) OR (West Africa) OR (Central Africa) OR (Western Sahara) | 0 |
| (Expenses) AND | (Hypertension) AND | (Sub Saharan Africa) OR (Angola) OR (Benin) OR (Botswana) OR (Burkina Faso) OR (Burundi) OR (Cabo Verde) OR (Cameroon) OR (Central African Republic) OR (Chad) OR (Comoros) OR (Congo) OR (Democratic Republic Congo) OR (Cote d’Ivoire) OR (Ivory Coast) OR (Djibouti) OR (Equatorial Guinea) OR (Eritrea) OR (Ethiopia) OR (Gabon) OR (Gambia) OR (Ghana) OR (Guinea) OR (Guinea-Bissau) OR (Kenya) OR (Lesotho) OR (Liberia) OR (Madagascar) OR (Malawi) OR (Mali) OR (Mauritania) OR (Mauritius) OR (Mozambique) OR (Namibia) OR (Niger) OR (Nigeria) OR (Reunion) OR (Rwanda) OR (Sao Tome and Principe) OR (Senegal) OR (Seychelles) OR (Sierra Leone) OR (Somalia) OR (South Africa) OR (Sudan) OR (Swaziland) OR (Tanzania) OR (Togo) OR (Uganda) OR (Zambia) OR (Zimbabwe) or (East Africa) OR (Middle Africa) OR (Southern Africa) OR (West Africa) OR (Central Africa) OR (Western Sahara) | 5 |

Total: 751
